# Medial Humeral Epicondyle Fracture Incarcerated Into the Elbow Joint in an Adolescent Patient With Ulnar Nerve Palsy

**DOI:** 10.7759/cureus.34502

**Published:** 2023-02-01

**Authors:** Rishab C, Dilipkumar Naidu, Thirumalai G, Aravinthan D R

**Affiliations:** 1 Orthopaedic Surgery, SRM Institute of Science and Technology, Chennai, IND; 2 Orthopaedics, SRM Institute of Science and Technology, Chennai, IND

**Keywords:** pediatric elbow fractures, elbow fractures, ulnar nerve palsy, incarcerated fragment, medial humeral epicondyle fracture

## Abstract

Medial humeral epicondyle fractures are seen in about one-fourth of all elbow fractures in the pediatric population. Though it seems to be a common occurrence, there is still controversy existing on the treatment aspects to date. Among these fractures, about one-fourth are seen incarcerated into the elbow joint and this is managed surgically. This is a case report of an adolescent male who had a medial epicondyle fracture of the humerus with the fracture fragment incarcerated into the elbow joint, and the patient also had ulnar nerve palsy, He was surgically treated with screw fixation and had an uneventful intra-operative and post-operative period.

## Introduction

Medial humeral epicondyle fractures are more common in paediatric populations than in adults. Though these fractures have been discussed extensively in the literature, there is still controversy in the treatment aspects of whether to go for conservative or surgical management [1-4]. The treatment aspect is very significant as an improper reduction in the paediatric population can lead to serious complications like growth arrest, which might further lead to deformity around the elbow in later life, as it is still a growing region of the elbow. The velocity of injury, displacement of the fracture, patient’s age and duration of initiation of treatment are very important for clinical outcomes.

The ulnar nerve passes behind the medial epicondyle of the humerus. In most cases, any injury around the medial condyle of the humerus affects the ulnar nerve. Hence, it is very important to assess the distal neurological status of any injury around the elbow, especially on the medial aspect.

This is a case report of a 17-year-old male with a medial humeral epicondyle fracture incarcerated into the elbow joint with ulnar nerve palsy treated promptly. Since the literature supports surgical management in the case of incarcerated fragments into the joint [3,4], this patient was treated surgically with an uneventful postoperative period.

## Case presentation

A 17-year-old male studying in school with right-hand dominance presented with severe pain and swelling over his left elbow for two days. The patient had a history of a skid and fall from a bicycle, sustaining an injury to the left elbow. He had fallen on an outstretched hand while landing on the palm. He was immediately taken to a native bone setter and only splinting was done. The parents brought the patient to us for a second opinion. We removed the splint and checked the elbow. The patient had severe pain from the time of the fall, which was acute in onset and aching type of pain. The pain was severe in intensity and was associated with diffuse swelling and restriction of movements. He had a deformity in his ring and little fingers. There was hyperextension at the metacarpophalangeal joint and flexion at the interphalangeal joints, which is termed 'claw hand deformity'. The sensation along the ring and little finger was reduced. He did not have any wounds on the skin and he was able to move his other fingers normally. His wrist movements caused pain at the elbow.

**Figure 1:**
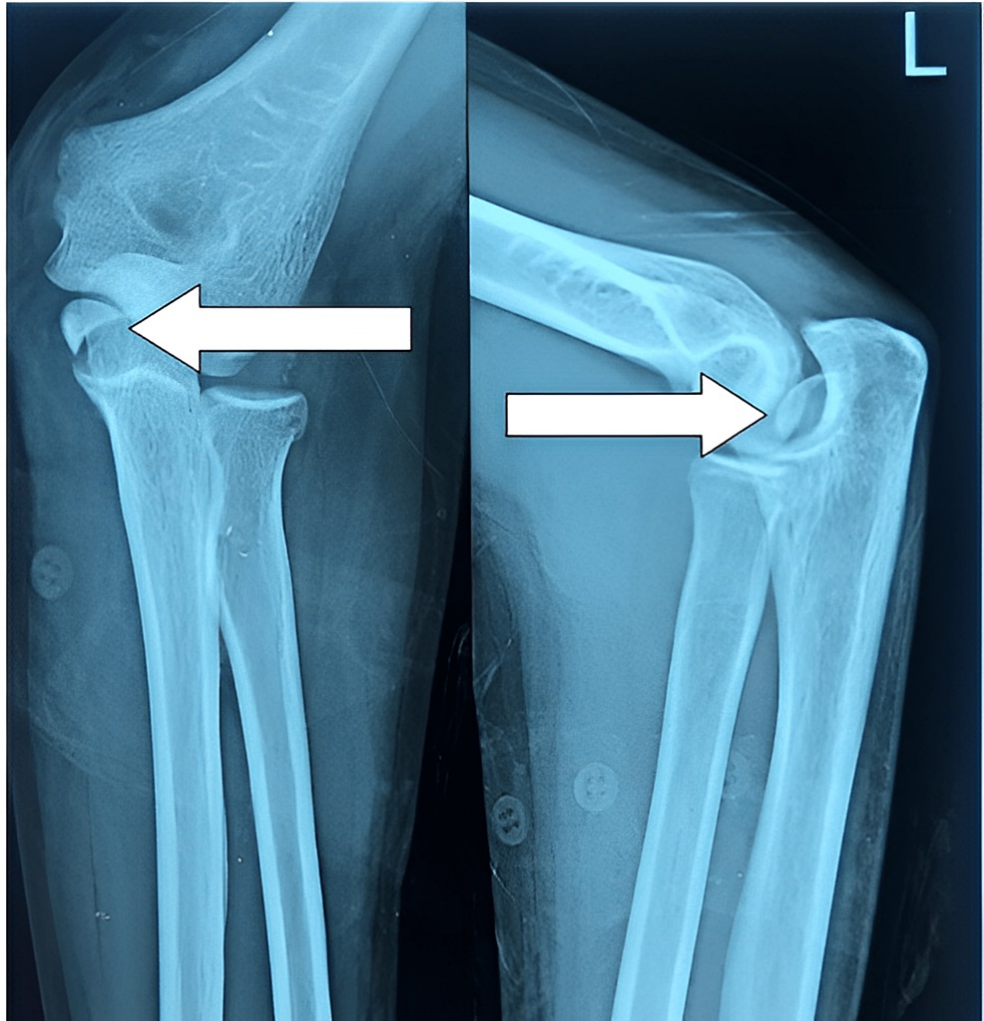
X-ray of left elbow showing the fractured medial humeral condyle (white arrow) into the joint with ulno-humeral subluxation

On examination, the patient was observed to have the arm by the side of the chest, elbow in flexion of 70˚, and forearm in supination. There was diffuse swelling of the left elbow. There was no warmth and tenderness was diffuse; the patient was not willing for further palpation due to pain. The patient had restrictions of movement due to pain. Apart from ulnar nerve palsy mentioned earlier, there was no other distal neuro-vascular deficit. Plain X-rays of the left elbow in both anteroposterior and lateral views showed a medial humeral epicondyle fracture incarcerated into the elbow joint giving the appearance of elbow subluxation (Figure 1).

**Figure 2:**
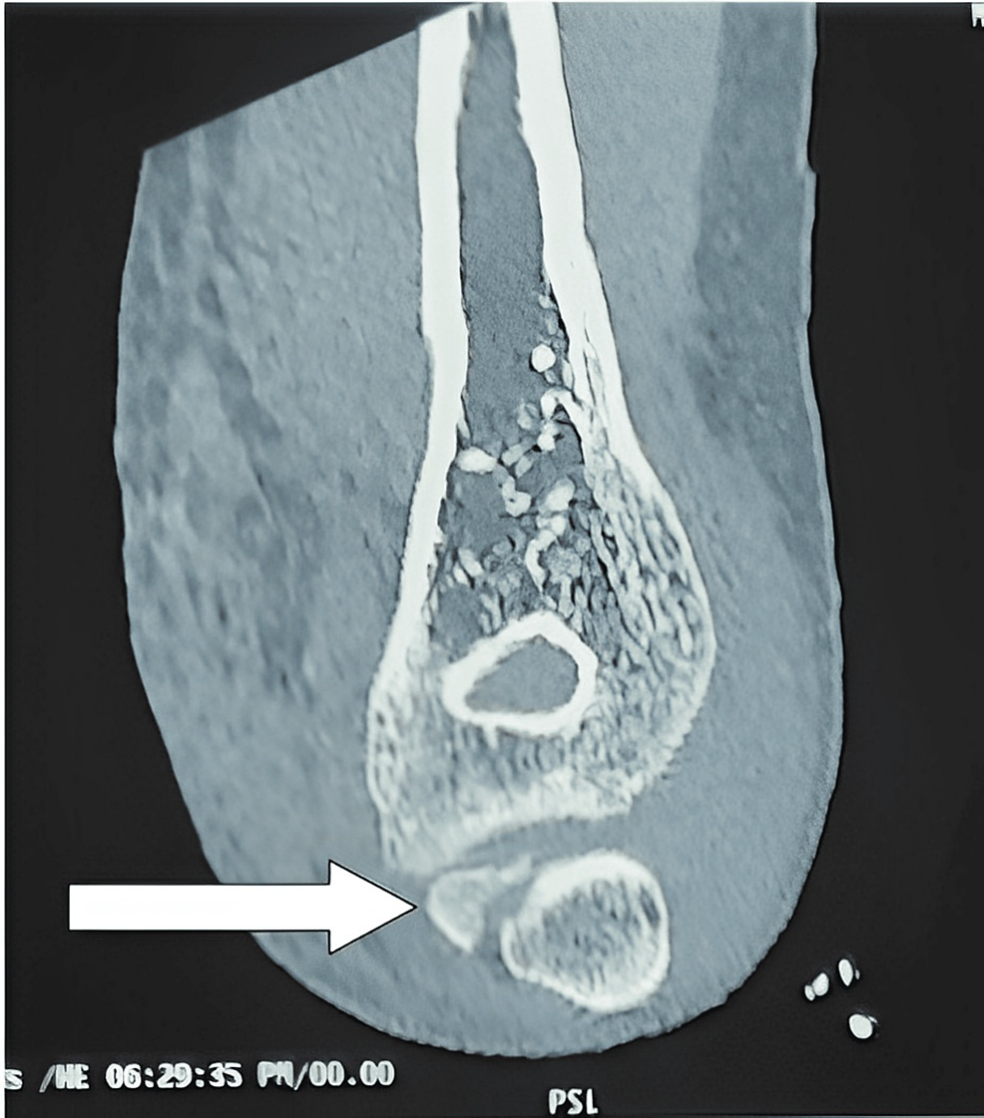
CT of left elbow (coronal image) showing the fragment (white arrow) into the joint

**Figure 3:**
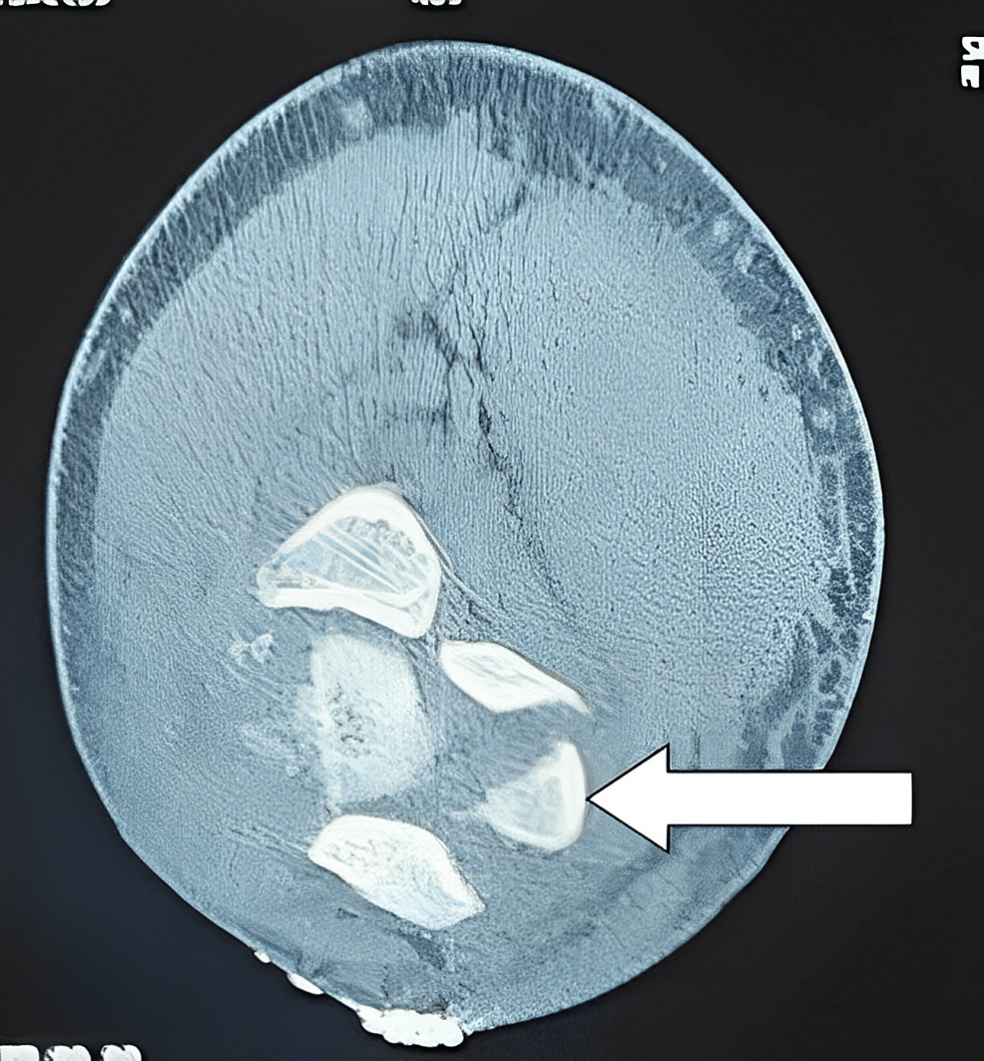
CT of left elbow (axial image) showing fractured fragment (white arrow) into the joint.

CT scan was also done to assess the incarcerated fragment and to confirm X-ray findings (Figures 2-3)

The patient was advised to undergo open reduction with screw fixation. The parents were hesitant about surgery and were initially unwilling for the same. Hence, he was given splinting with an above-elbow plaster slab. The patient, together with his parents, came to our outpatient department, two days later, with a willingness for surgery. We then proceeded with proper consent and guarded prognosis for the ulnar nerve recovery, before he was evaluated for surgery.

**Figure 4:**
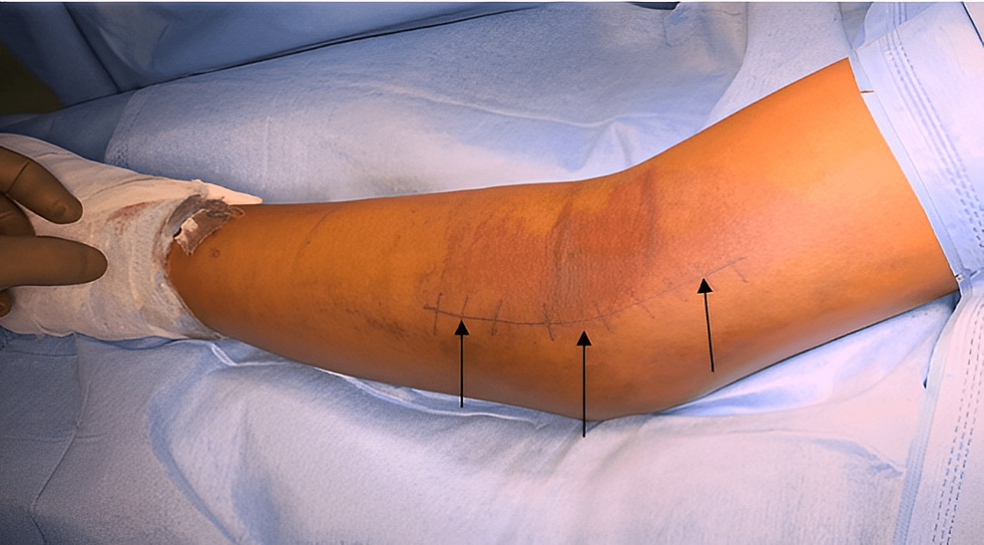
The affected elbow showing the line of skin incision (black arrows) for Hotchkiss approach.

Under general anaesthesia, the patient was positioned in supine with the left upper limb in supination on the arm board. The skin was marked for Hotchkiss medial approach for elbow (Figure 4).

**Figure 5:**
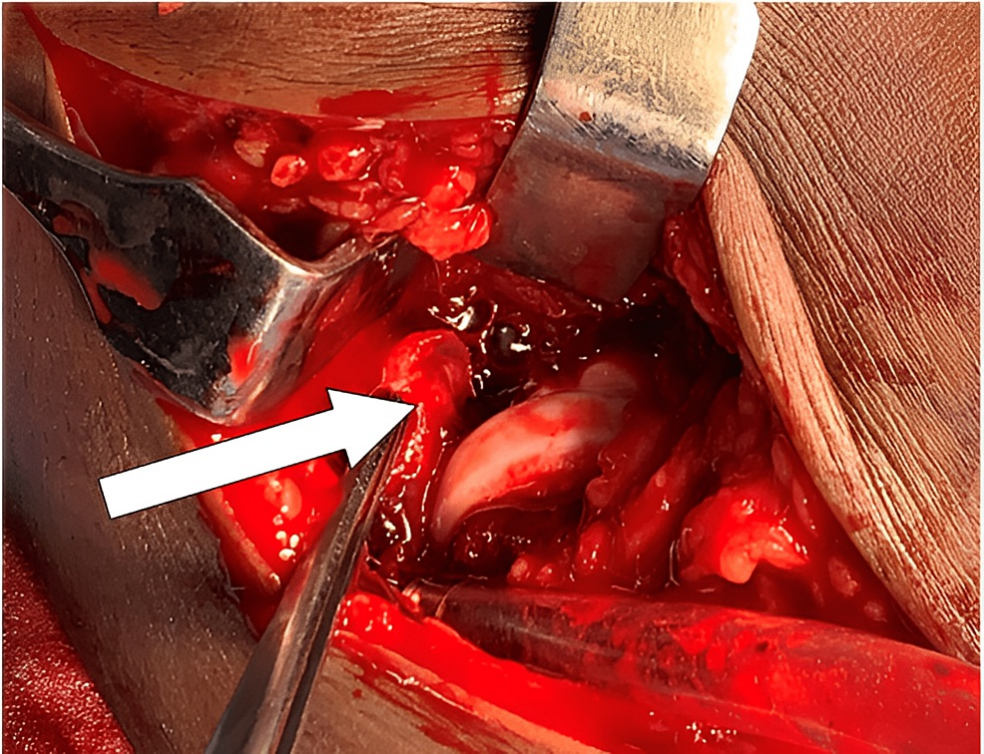
Fractured fragment (white arrow) seen in the elbow joint.

The dissection was deepened. The ulnar nerve was identified, traced a few centimetres proximally as well as distally, and the nerve was protected. The capsule of the elbow joint was dissected and the fracture fragment was visualised in the joint (Figure 5).

**Figure 6:**
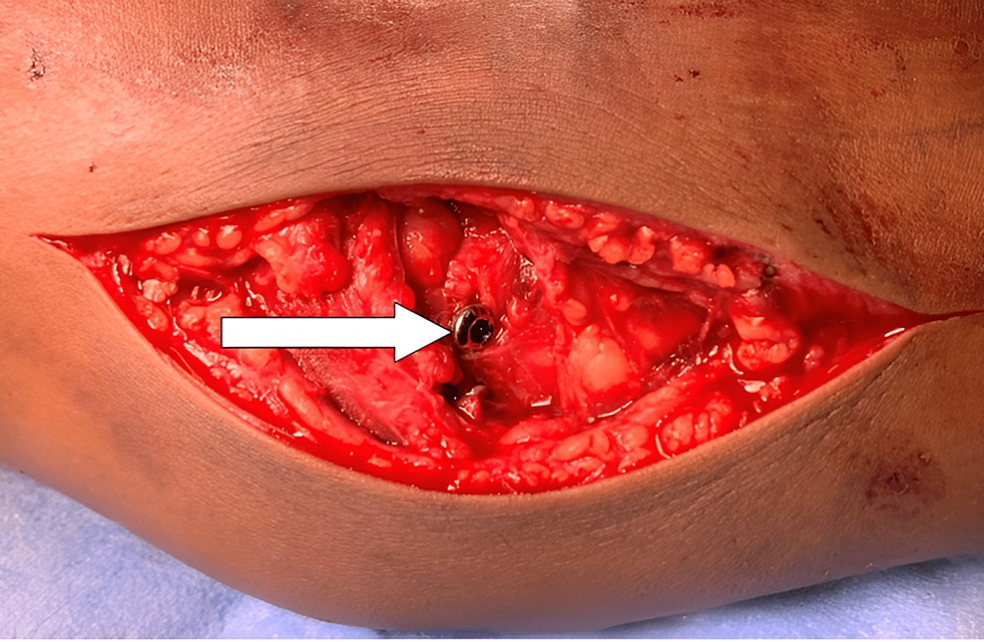
The fragment was reduced and stabilized with a cannulated cancellous screw (white arrow).

As soon as the incarcerated medial humeral condylar fragment was removed, the elbow joint reduced. Then the fragment was freshened and stabilised to medial humeral condyle with a guide wire under C-arm guidance. The fragment was then fixed with a cannulated cancellous screw and a washer (Figure 6).

**Figure 7:**
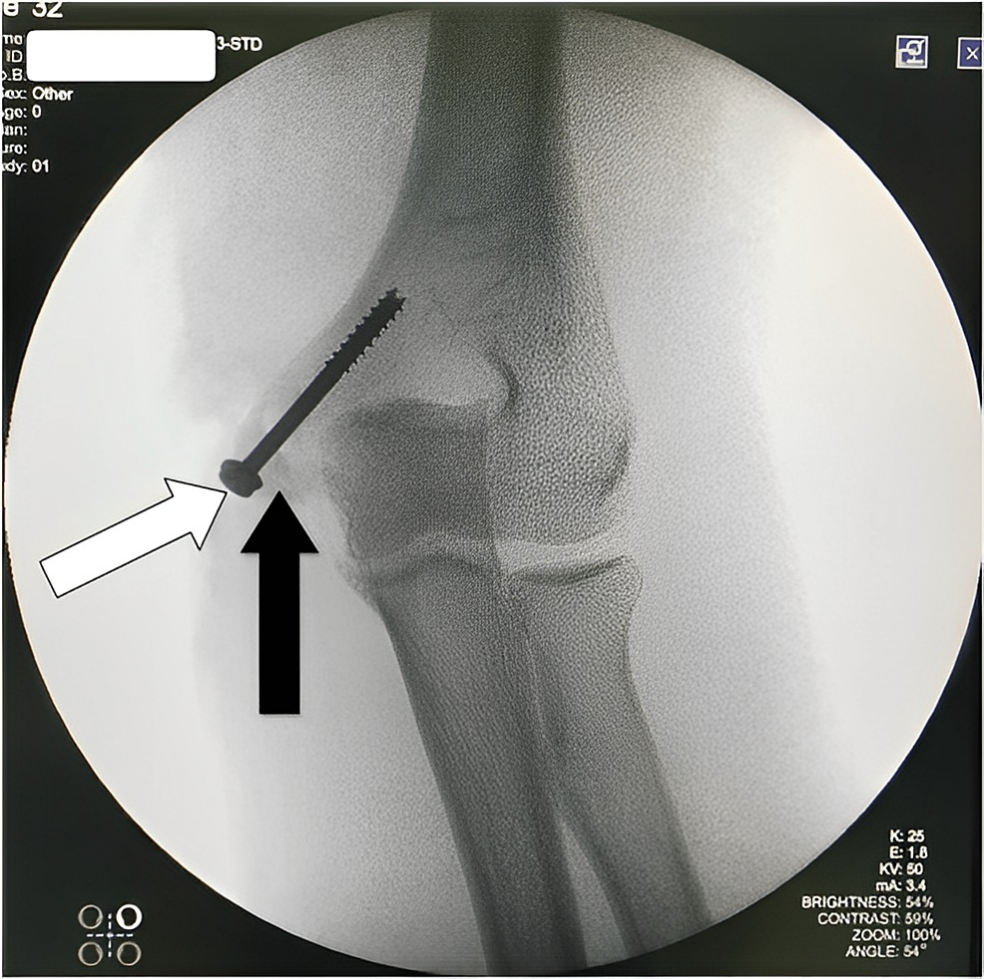
The screw (white arrow) is holding the fractured fragment (black arrow) in its place under C-arm.

**Figure 8:**
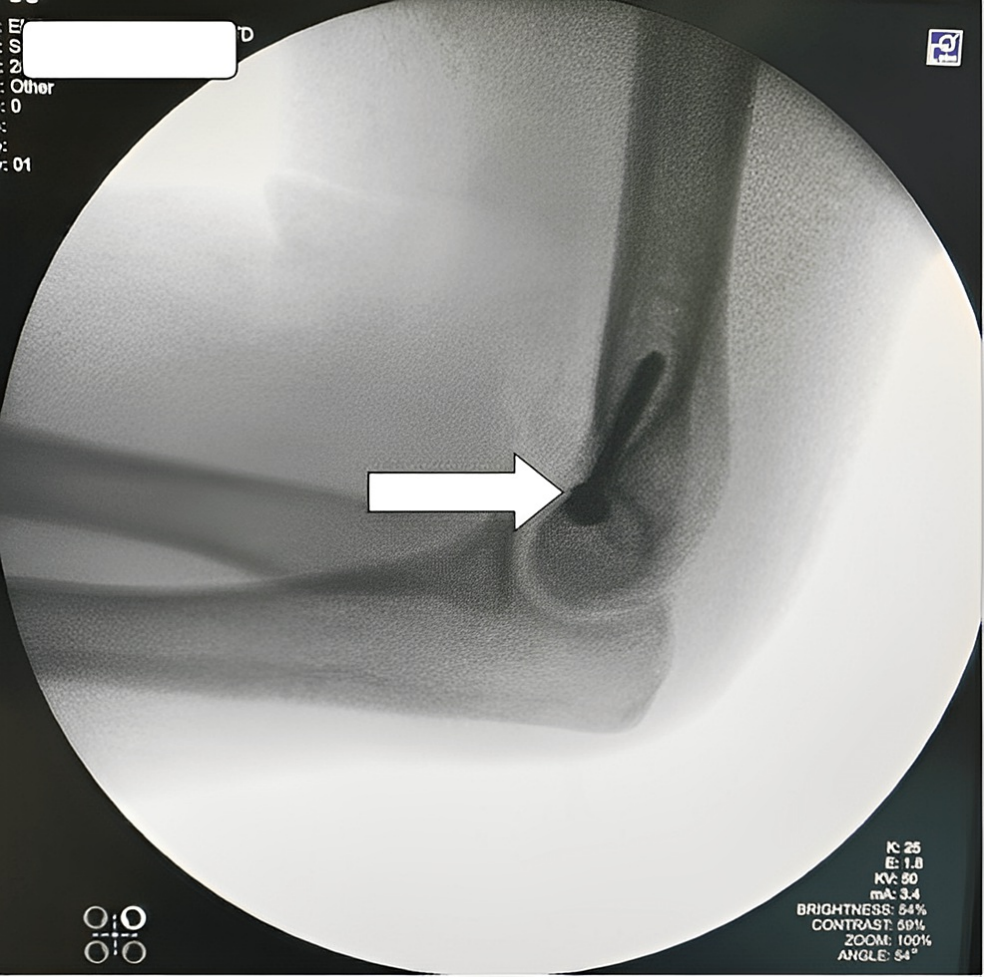
Lateral view of the elbow showing the screw (white arrow) in the proper position

Reduction was found to be satisfactory under C-arm (Figures 7-8).

**Figure 9:**
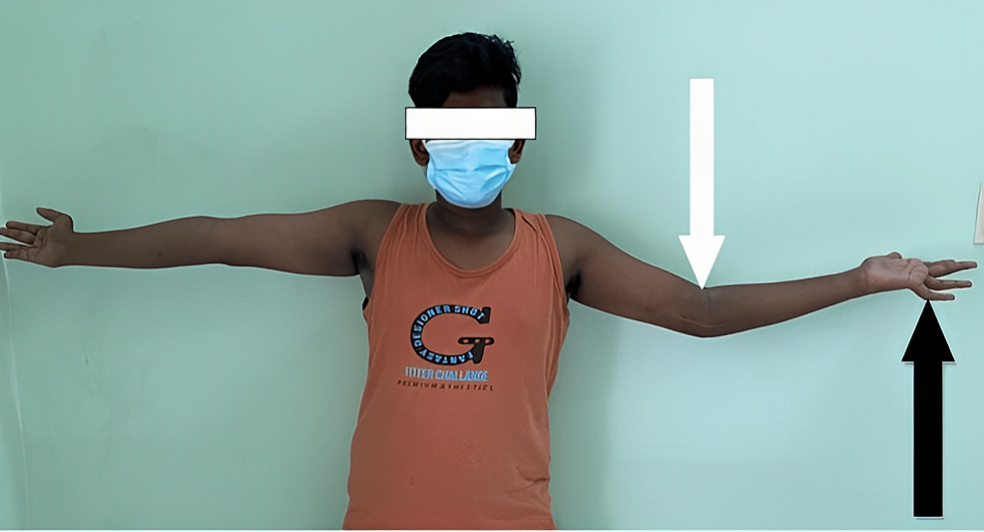
Clinical picture of the patient showing elbow movements (white arrow) with extension lag of 20 degrees and ring and little fingers (black arrow) of left hand, showing claw hand deformity.

**Figure 10:**
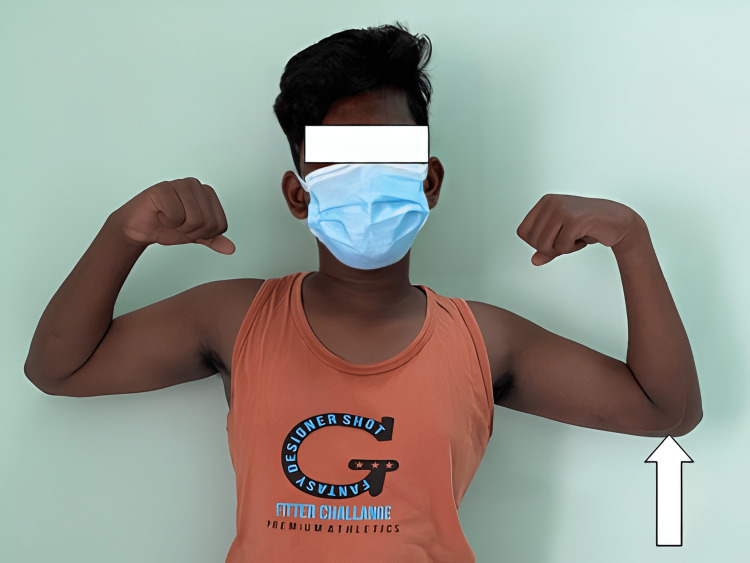
The affected left elbow (white arrow) had flexion up to 110 degrees which appears to be equal to that of the normal right elbow.

The elbow was found to be stable. The wound was washed and closed in layers and the joint was immobilised on an above-elbow plaster slab. The sutures were removed 12 days postoperatively. The above-elbow plaster slab was removed after three weeks, and active elbow mobilisation was started. Since there was a history of native bandaging, the patient was started on oral indomethacin preoperatively, administered for three weeks after surgery, in view of preventing heterotopic ossification. The patient was followed up for three months, and he did not come for further follow-up after that. At the third-month follow-up, he had an extension lag of 20˚ (Figure 9) and flexion of 20˚-110˚ (Figure 10) at the elbow. The wrist movements were full but he still had clawing of the little and ring fingers (Figure 9).

## Discussion

Medial humeral epicondyle fractures are seen in about 15-20% of all elbow fractures in the pediatric population [4-6]. Although many authors have explained various treatment modalities, there is still debate between conservative and surgical management for medial epicondyle fracture of the humerus [1-4]. Many authors support surgical management as there is a high chance of bony non-union with conservative management [2]. While bony non-union is seen in some patients, the functional disability remains statistically insignificant [7]. However, surgery can be cosmetically unacceptable and needs hardware removal [4]. The opinions regarding conservative treatment and surgery have also been explained with regard to the displacement of the fracture fragments. There are many ossification centres in the elbow joint, which ossifies at various stages of adolescent life, making it weaker compared to other joints. There are three possible mechanisms [8] with which the medial condyle of the humerus can get fractured: (i) A fall on an outstretched arm, with the elbow forced into valgus, (ii) A fall on the point of the elbow (apex of the flexed elbow), with the olecranon driving the medial condyle proximally and medially, and (iii) An avulsion fracture due to violent contraction of the flexor and pronator muscles that attach to the medial epicondyle.

Despite the debate between conservative and surgical management for medial epicondyle fractures, all studies mandate surgical management for incarcerated fragments into the elbow joint as definitive management, as in our case. Some authors have taken the displacement of the fracture fragment as a criterion to decide the treatment [3]. In some studies, for displacements less than 2 mm, conservative management is regarded as ideal and displacements more than 5 mm need surgical management [4]. In order to assess the displacement, CT is required for better accuracy as plain X-rays (anterioposterior and lateral views) alone cannot pick up the accurate displacement and size of the fragment [9]. Few studies are against CT due to the ionising radiation affecting the young population and also due to cost factors [5,10].

Another view on X-ray is the internal oblique view lateral radiograph in about 60˚ and 45˚ rotations to assess the displaced fragment [5]. But even this view has been found to have low inter and intra-observer variability. A fourth view, the distal humeral axial view, has been shown to have better variability compared to the standard anterioposterior, lateral, and internal oblique views [10]. The axial image is taken by positioning the central ray above the shoulder at 15-20˚ from the long axis of the humerus centred on the distal humerus. As per this view, for displacements <10mm, mean error in measurements 1.5±1.1mm, and >10mm displacements, the error in measurement was 0.8±0.7mm. This was significantly less compared to standard anterioposterior, lateral, and internal oblique views. If still in doubt, some authors have suggested the use of ultrasonography (USG) of the elbow for the assessment of fractures as most of the fragments in the paediatric age group remain un-ossified [11]. USG can pick up posterior fat pad sign, and lipo-hemarthrosis with sensitivity and specificity up to 100% and 92%, respectively.

While surgery is preferred by most authors, there is controversy over the choice of implants. Some authors prefer Kirschner wires (K-wires) in younger patients and screw in older age groups [12]. Screws may cause soft tissue irritation and demands early hardware removal. Moreover, their study has shown that the use of a washer also makes implant removal complicated [9]. After surgery, all patients definitely need long arm splinting for up to three to four weeks. Some authors have mentioned that if the immobilisation is prolonged, it might lead to more stiffness of the elbow with a restriction of extension of 20-25˚ [3,6]. Loss of extension is better tolerated than a loss of flexion [2,3], so immobilisation for up to four weeks seems acceptable. After immobilisation, active movement is started as tolerated.

## Conclusions

Surgery is the only definitive management for medial humeral epicondyle fracture incarcerated into the elbow joint. Conservatively, the fragment cannot be reduced. Regarding fractures of medial humeral epicondyle (without incarceration into the elbow joint), a large randomised control study is necessary to explore the definitive management.
